# *In vitro* Evaluation of Porous borosilicate, borophosphate and phosphate Bioactive Glasses Scaffolds fabricated using Foaming Agent for Bone Regeneration

**DOI:** 10.1038/s41598-018-22032-2

**Published:** 2018-02-27

**Authors:** E. P. Erasmus, R. Sule, O. T. Johnson, J. Massera, I. Sigalas

**Affiliations:** 1 African Material Science and Engineering Network (A Carnegie-IAS RISE Network), Johannesburg, South Africa; 2 University of the Witwatersrand School of Chemical and Metallurgical Engineering, Johannesburg, South Africa; 3 DST/NRF Centre of Excellence in Strong Materials, University of the Witwatersrand, Johannesburg, South Africa; 4 University of Namibia Department of Mining and Metallurgical Engineering, Ongwediva, Namibia; 5 Tampere University of Technology BioMediTech institute and Faculty of Biomedical Sciences and Engineering, Tampere, Finland

## Abstract

In this work, glasses within the borosilicate borophosphate and phosphate family were sintered into 3D porous scaffolds using 60 and 70 vol. % NH_4_(HCO_3_) as a foaming agent. All scaffolds produced remained amorphous; apart from one third of the glasses which crystallized. All produced scaffolds had porosity >50% and interconnected pores in the range of 250–570 µm; as evidenced by µCT. The *in-vitro* dissolution of the scaffolds in SBF and changes in compression were assessed as a function of immersion time. The pH of the solution containing the borosilicate scaffolds increased due to the typical non-congruent dissolution of this glass family. Borophosphate and phosphate scaffolds induced a decrease in pH upon dissolution attributed to the congruent dissolution of those materials and the large release of phosphate within the media. As prepared, scaffolds showed compressive strength of 1.29 ± 0.21, 1.56 ± 0.63, 3.63 ± 0.69 MPa for the borosilicate, borophosphate and phosphate samples sintered with 60 vol. % NH_4_ (HCO_3_), respectively. Evidence of hydroxyapatite precipitation on the borosilicate glass scaffolds was shown by SEM/EDS, XRD and ICP-OES analysis. The borophosphate scaffolds remained stable upon dissolution. The phosphate scaffolds were fully crystallized, leading to very large release of phosphate in the media.

## Introduction

As human life span continues to rise and degenerative bone diseases become more common, especially in elderly people, the need for synthetic biomaterials for the repair and regeneration of bone is becoming increasingly important^1^. Currently, off the shelfs porous scaffolds are in demand due to their architectural structure which have shown to be crucial in the bone healing process. These scaffolds are not only required to mimic the morphology, structure and function of bone, but should also: (1) be biocompatible (not toxic) and should promote cell adhesion and proliferation; (2) exhibit mechanical properties after *in vitro* tissue culture which are comparable to those of the bone to be replaced; (3) have a porous three-dimensional (3D) architecture to allow cell proliferation, vascularization, and diffusion of nutrients between the cells seeded within the matrix and the surroundings; (4) degrade, at a rate that matches the production of new bone, into non-toxic products that can be easily resorbed or excreted by the body; (5) be processed economically into anatomically relevant shapes and dimensions and be sterilized for clinical use^2^.

Among other biomaterials; bioactive glasses have shown some ideal properties for use as scaffold materials. These glasses (i) can be tailored to be bioactive (osteoconductive and osteoinductive) and (ii) their degradation rate can be tailored to match the growth of the new tissues^3^. The bioactivity of a glass is often linked to its ability to form hydroxyapatite (HA) on its surface, with structure and composition similar to that of the natural bone mineral. This layer favors attachment of soft/hard tissues^4^; resulting in biological fixation. However, the initially developed, and FDA approved, bioactive glasses were found to possess a structure and a crystallization mechanism inhibiting proper sintering of 3D scaffolds while maintaining the materials amorphous nature^5–8^. As reported by Bellucci *et al*. the impact of crystallization on the glass’ bioactivity is contradictory^9^. While in some cases the glass bioactivity was reported to be suppressed; in some other cases it was only slowed down; and this was reported in both silicate and phosphate-based bioactive glasses^6,10–12^. Overall; the bioactivity of the glass-ceramic was related; to some extent; to the remaining glassy phase and/or to the solubility of one of the crystal phase^6,10^.

Researchers have focused part of their attention to develop bioactive glasses that could be sintered into porous structure without undergoing crystallization. This is the case for instance of the glass 13–93, developed by Brink *et al*., which demonstrate thermal properties enabling the sintering of powder without adverse crystallization^13,14^. However, similarly to glass-ceramic the reaction/dissolution rate is greatly reduced compared to more traditional bioactive glass such as 45S5 (Bioglass^®^) or S53P4 (BonAlive^®^). While in some applications this may not be disadvantageous, (in lace or in patient with slow tissue growth), for other applications fast reaction rates would be beneficial. Furthermore, uncontrolled crystallization and unknown amount of remaining amorphous phase may lead to unpredictable dissolution rate and mechanism. Therefore, the focus has shifted to other glass types, i.e., phosphate and borate glasses. Phosphate and borophosphate glasses typically possess more advantageous thermal processing window than silicate glasses as demonstrated by its ability to be drawn into fibers without any crystals formation from either bulk or melt^15,16^. Borate glasses were also found to possess hot forming domain (range of temperature at which the samples can be shaped without adverse crystallization, typically defined as the difference between T_x_ the onset of crystallization and T_g_ the glass transition temperature) well above their silicate glass counterpart as demonstrated by Rahaman *et al*.^17^. However, glasses with large boron content were found to reduce, compared to silicate bioactive glasses, the amount of cells attaching and proliferating at the surface of the glass surface; in both static and dynamic cell culture test^17^. One should also note that, despite their lower surface affinity to cell attachment and proliferation, a small animal study was found very promising towards fast and complete conversion of borate glasses into new bone^18^.

A number of methods have been used to fabricate three-dimensional (3D) scaffolds of bioactive glasses including foam replication; direct foam and sol-gel process. Previous work has shown that foam replication can be used to fabricate 45S5 and 13–93 bioactive scaffolds. This method yields high porosity with large pores with a good chance of permeability, however due to cracking of the struts during pyrolysis, the mechanical properties of the scaffolds are generally poor^19^. Bioactive glass scaffolds created through the sol-gel process have also attracted interest in tissue engineering applications. One such scaffold is the 70S30C bioactive glass scaffold^20^. Scaffolds obtained through this technique often have a high surface area (100–200 m^2^/g) due to the inherent porosity of the process, as a result, these scaffolds degrade and convert faster to HA than scaffolds processed from melt derived glass with the same composition. The main drawbacks lies in their poor mechanical properties and the time taken to produce sol-gel glasses.

Recently, glasses within the borosilicate, borophosphate and phosphate compositions were sintered into porous scaffolds by, simple heat treatment of fine glass particles (<38 µm) mixed with ammonium carbonate as porogen^21^. The borosilicate glass was tailored to favor glass conversion into HA while increasing the hot forming domain^21^. The phosphate glass label PSr40 was found to promote gingival fibroblasts^22^. However this glass was found to crystallize readily during scaffold processing partly due to their rapid surface crystallization^12,21^. To overcome the fast crystallization of the phosphate glass, born was introduced. The subsequently developed borophosphate possess a larger hot working domain and a reduced dissolution rate^15^.

Therefore; in this study borosilicate glass (S53B50), borophosphate glass (P40B10) and a phosphate glass (PSr40) were sintered into porous scaffolds using 60 and 70 vol. % NH_4_(HCO_3_) as foaming agent and as reported in^21^. The obtained scaffolds were immersed in simulated body fluid (SBF) in order to assess the dissolution of the scaffolds as well as their ability to precipitate a HA reactive layer. The mechanical properties of the scaffolds was measured in compression, as a function of immersion time.

## Results

### Scaffold characterization

The porosity and average pore size of the as-prepared scaffolds was determined using micro-computed tomography (µCT) and the obtained data are reported in^21^. From the results, as expected, the average pore size and porosity increased with increasing the NH_4_ (HCO_3_) content from 60 to 70 vol. %. As previously discussed the pore size and overall porosity are within the acceptable limits for bone tissue applications.

**Figure 1:**
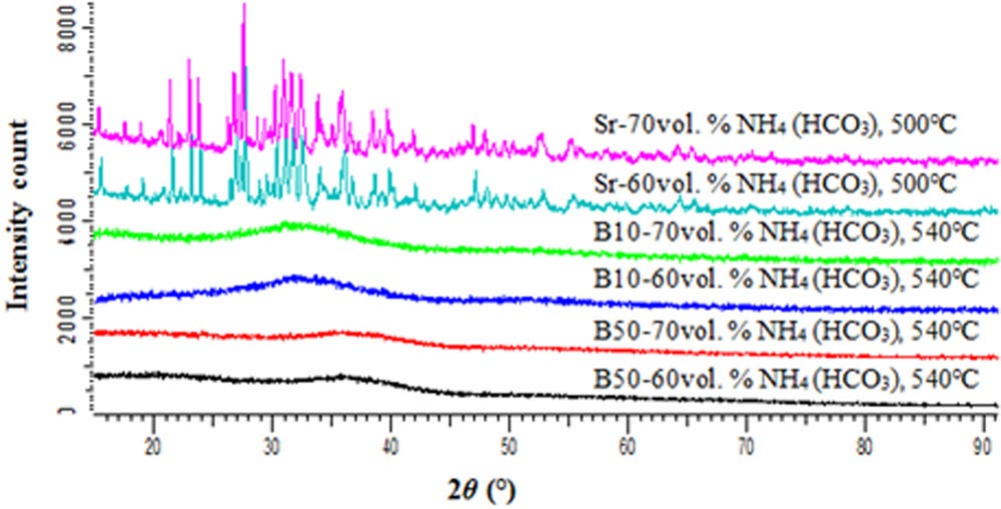
XRD patterns of the porous glass scaffolds for S53B50, P40B10 and PSr40.

XRD analysis was conducted on the as sintered scaffolds to determine if any crystallization had occurred. Glass S53B50 and P40B10 were found to maintain their amorphous nature during sintering (Fig. 1), whereas glass PSr40 endured pronounced crystallization at all temperatures leading to sintering. While the goal was to maintain the amorphous nature of the scaffolds, these crystallized scaffolds were studied to better understand the impact crystallization has on the dissolution of phosphate bioactive glasses.

### *In vitro* dissolution in simulated body fluid (SBF)

**Figure 2:**
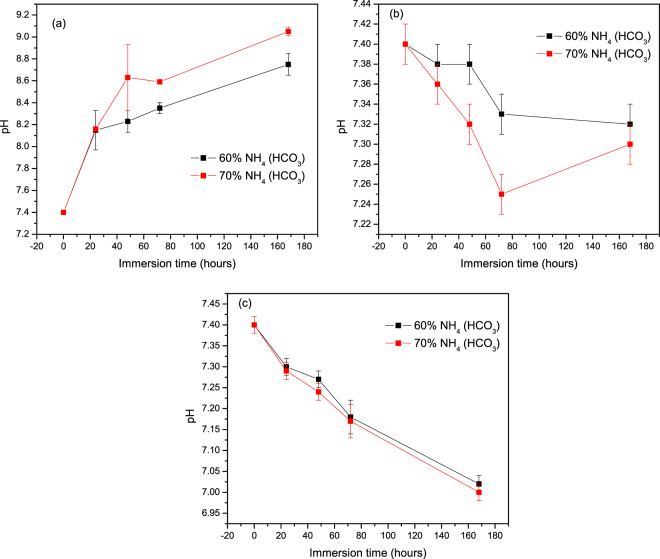
pH of SBF as a function of immersion time upon immersion of the (**a**) S53B50 (**b**) P40b10 and (**c**) PSr40.

Figure 2a,b and c presents the average pH values of the SBF solutions containing glass scaffolds of S53B50, P40B10 and PSr40, respectively, as a function of immersion time and for varying contents of NH_4_ (HCO_3_) at 37 °C. The pH value of the SBF before immersion is 7.4.

The pH of the solution containing the borosilicate bioactive glass scaffold (Fig. 2a) increased with increasing immersion time, which increased to pH 9 at 168hrs. Additionally, the pH values of the scaffolds with 70 vol. % NH_4_ (HCO_3_) was found to be higher than that of the scaffolds with 60 vol. % content.

The pH results for glass P40B10 and PSr40 scaffolds are shown in Fig. 2b and c, respectively. The pH of the SBF solution decreased with respect to immersion time; in contrast to the scaffolds of glass S53B50. However, they could still be regarded as being relatively neutral as they don’t go below pH 7. As observed with the glass scaffolds of S53B50 the changes in pH of the scaffolds with 70 vol. % NH_4_(HCO_3_) were more pronounced than those with 60 vol. %.

**Figure 3:**
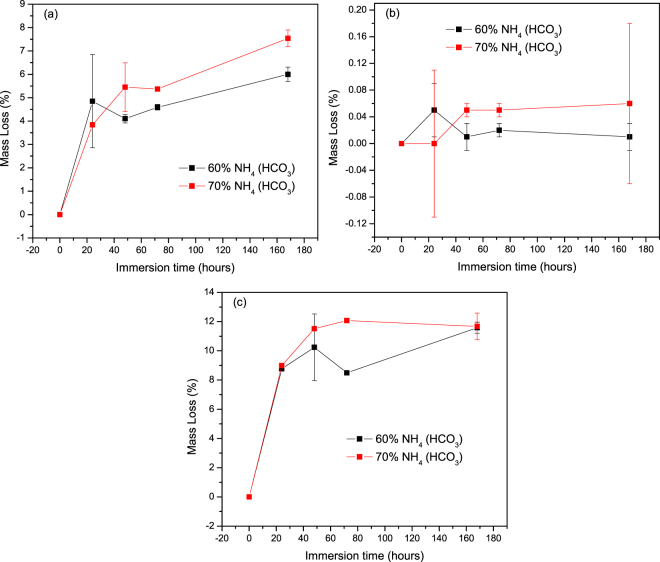
Mass loss (%) as a function of immersion time for scaffolds of (**a**) S53B50 (**b**) P40B10 and (**c**) PSr40.

The mass loss, which is also an indication of the material dissolution, was also recorded as a function of immersion times and are presented in Fig. 3a,b and c, for the S53B50, P40B10 and PSr40 scaffolds respectively. With increasing the immersion time, both the borosilicate (S53B50) and the phosphate (PSr40) glass exhibit a steep mass loss. The mass loss is rapid up to 24–48 h and then slows down. The borophosphate scaffolds (P40B10), however, presented little mass change.

**Figure 4:**
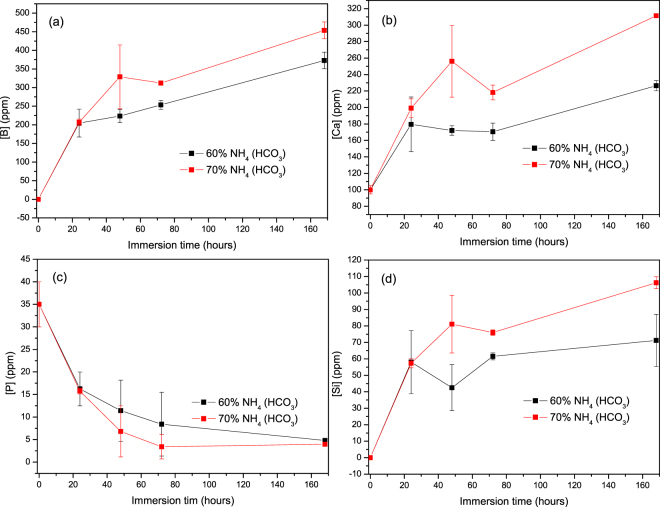
Ion concentration in the immersion solution of S53B50 for (**a**) B, (**b**) Ca, (**c**) P and (**d**) Si as a function of time.

After immersion, the ion concentration in the immersion solution was quantified using inductive coupled plasma. Figure 4 presents the (a) [B], (b) [Ca], (c) [P] and (d) [Si] ion concentration upon immersion of the borosilicate glass. Upon immersion, the [B]; [Ca] and [Si] ion concentration increased. The [P]; however was found to decrease with increasing immersion time. Up to 24 h of immersion; no significant differences in concentration could be measured between samples processed with 60 or 70 vol. % porogen. At longer immersion time in all ions concentration, except [P], are higher in the solution containing the samples prepared with 70% porogens. The consumption of [P] is higher in this samples compare to the one containing 60% foaming agent.

**Figure 5:**
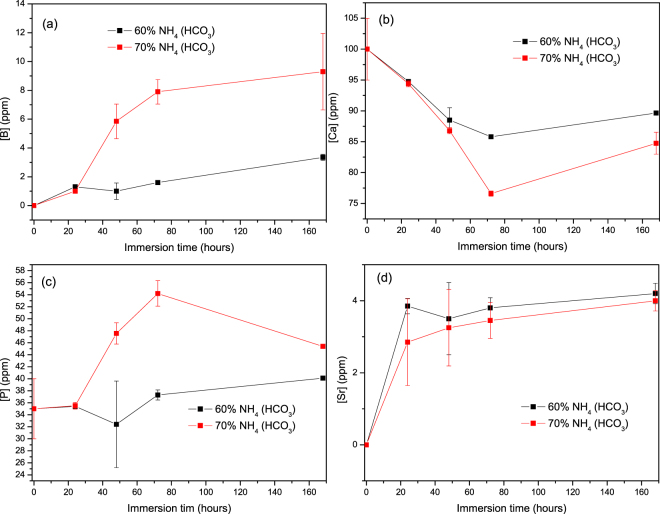
Ion concentration in the immersion solution of P40B10 for (**a**) B (**b**) Ca (**c**) P and (**d**) Sr as a function of time.

Figure 5 presents the (a) [B]; (b) [Ca]; (c) [P] and (d) [Sr] ion concentration upon immersion of the borophosphate glass. While a general increase in [B]; [P] and [Sr] can be seen; the [Ca] was found to decrease. As explained in Fig. 5; all variation are more pronounced when scaffolds containing 70 vol. % are immersed in SBF. It is also noteworthy to mention that concentration for [B], taken as an example is 30 times lower in the solutions containing the borophosphate glass (Fig. 5a) than in the solution containing the borosilicate glass (Fig. 4a).

**Figure 6:**
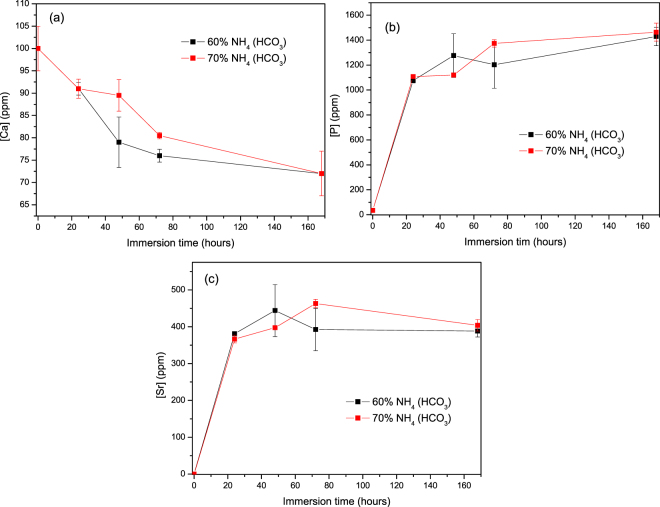
Ion concentration in the immersion solution of PSr40 for (**a**) Ca (**b**) P (**c**) Sr as a function of time.

Figure 6 presents the (a) [Ca]; (b) [P] and (c) [Sr] ion concentration upon immersion of the phosphate glass scaffolds. After 24 h in solution; the [P] and [Sr] increases and then remain almost constant for longer immersion time. The [Ca]; however; decreases during all imersion test. As opposed to the other tested materials; here no significant differences could be seen between the scaffolds produced with 60 or 70 vol. % of NH_4_(HCO_3_). It is noteworthy to point out that, the [P] content is ~1400 ppm at 1 week upon immersion of the phosphate scaffolds, whereas it never exceeds 50 ppm when immersing the borophosphate scaffolds. Similarly, [PSr40] reaches ~400 ppm when immersing phosphate scaffolds and only ~4 ppm when immersing borophosphate scaffolds.

### Phase and Microstructural analysis

#### XRD analysis

**Figure 7:**
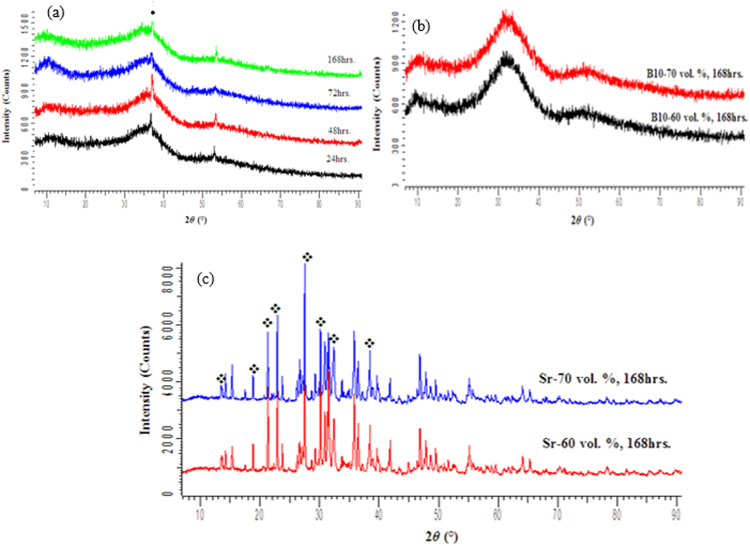
XRD traces of (**a**) S53B50–70 vol. % NH_4_ (HCO_3_) immersed in SBF for 24, 48, 72 and 168 hrs; (**b**) P40B10 and (**c**) PSr40 60 and 70 vol. % NH_4_ (HCO_3_) immersed in SBF for 168 hrs.

The XRD patterns of the S53B50 glass scaffolds after immersion in SBF for 24, 48, 72 and 168 hours are shown in Fig. 7a. The XRD patterns showed a major peak at 36° which could correspond to HA as reported in^23^. Normally this peak is found at about 32° when using Cu K_α_, however in this case the use of Co K_α_ causes a shift in the peak position. These peaks increased in intensity as the immersion time and porosity increased. However, as observed by^24^, the peak intensities were still well below those for a standard crystalline HA, this clearly indicates that the as-formed HA was incompletely crystallized or only weakly crystalline. In addition, it is possible that the low number and intensity of the diffraction peak is related to the small amount or low thickness of the reactive layer.

The XRD patterns for the treated scaffolds of glass P40B10 and PSr40 are shown in Fig. 7b and c respectively. The XRD results for both compositions (60 and 70 vol. %) were chosen at 168hrs to assess the formation of the reactive layer. The XRD pattern of P40B10 immersed for 168hrs remained identical to the untreated one. On the other hand, a phase difference was observed for the PSr40 scaffolds before and after immersion in SBF. This is evident by the reduction in peak intensity, formation of new peaks as well as the disappearance of some peaks.

#### SEM/EDX analysis

**Figure 8:**
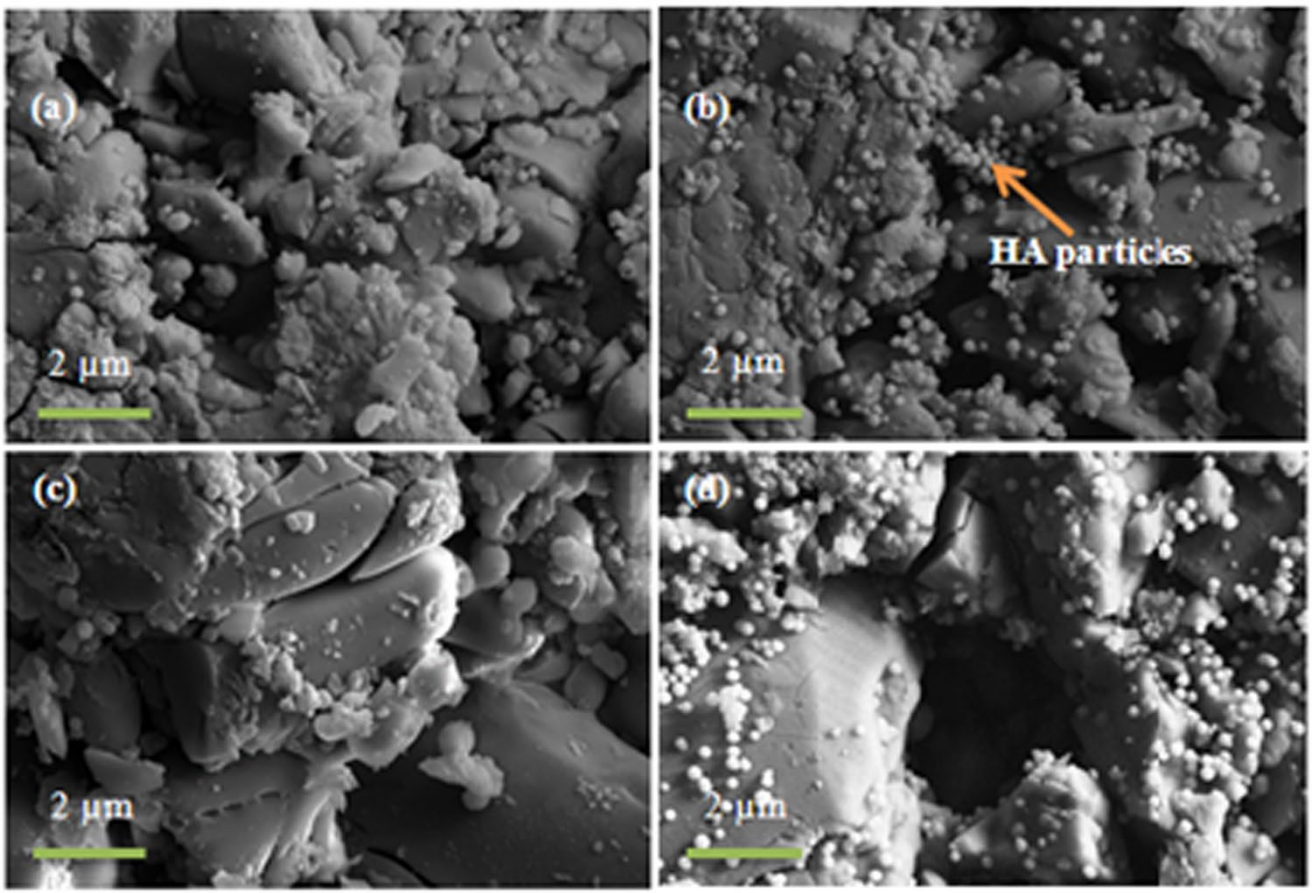
SEM images of the surface of S53B50–60 vol. % NH_4_ (HCO_3_) bioactive glass scaffolds after immersion in simulated body fluid for (**a**) 24 hr (**b**) 48 hr (**c**) 48 hr and (**d**) 168 hr.

Figure 8 presents SEM micrographs of the scaffold surfaces of S53B50 glass after immersion in SBF. The images show that upon immersion precipitation of spherical particles occurs. The formation of these globular particles were mainly observed on glass S53B50 in contrast to glass P40B10 and PSr40.

**Figure 9:**
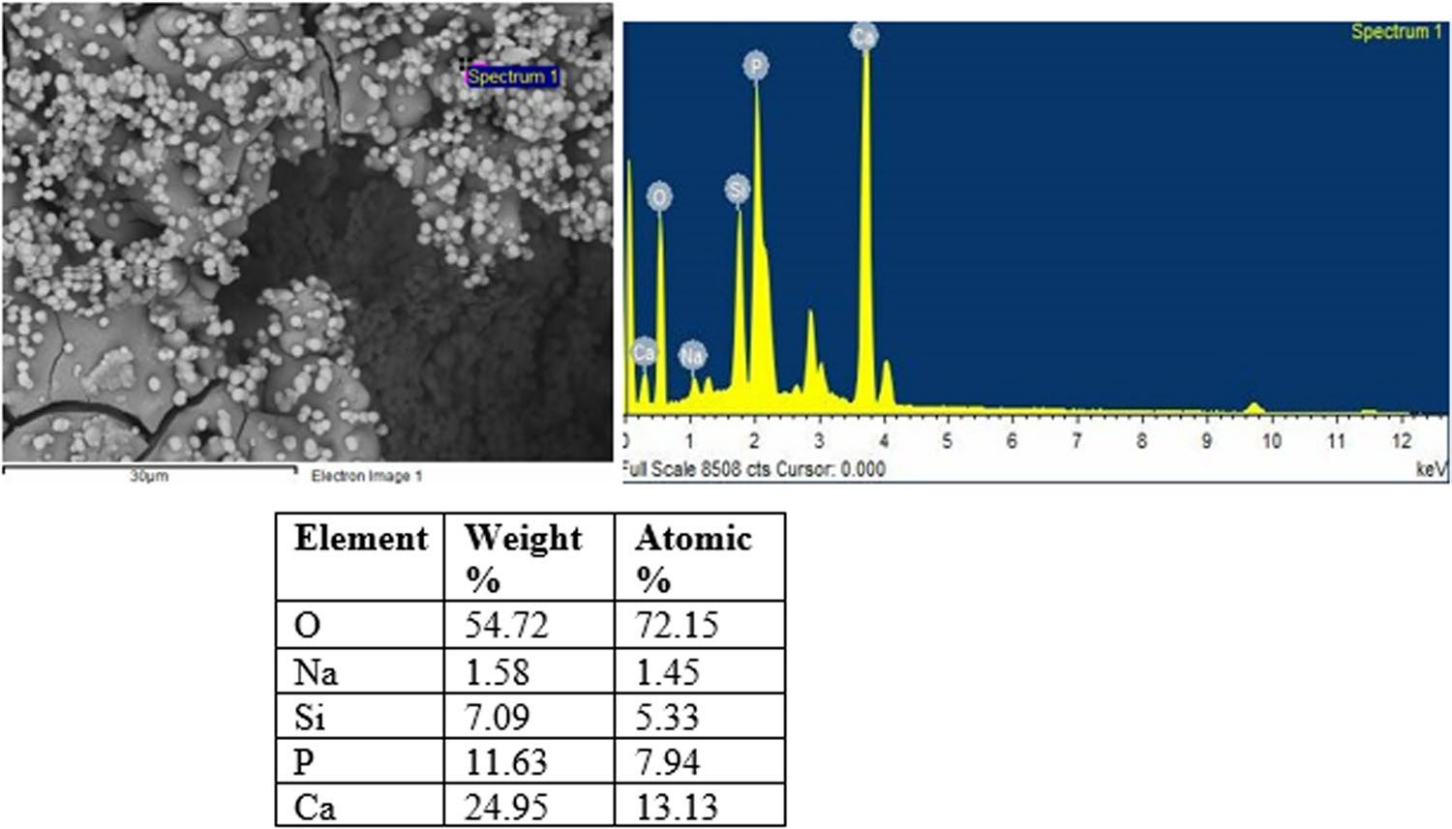
EDS spot analysis of HA particles on glass S53B50–70 vol. % NH_4_ (HCO_3_) after immersion in simulated body fluid for 24 hours.

Figure 9 presents the SEM image and corresponding EDX analysis of one of the particle of glass S53B50. From EDX analysis the Ca/P ratio is found to be 1.65. The presence of Si and Na come most likely from the glass underneath the particles. This is expected given the high penetration of X-ray.

**Figure 10:**
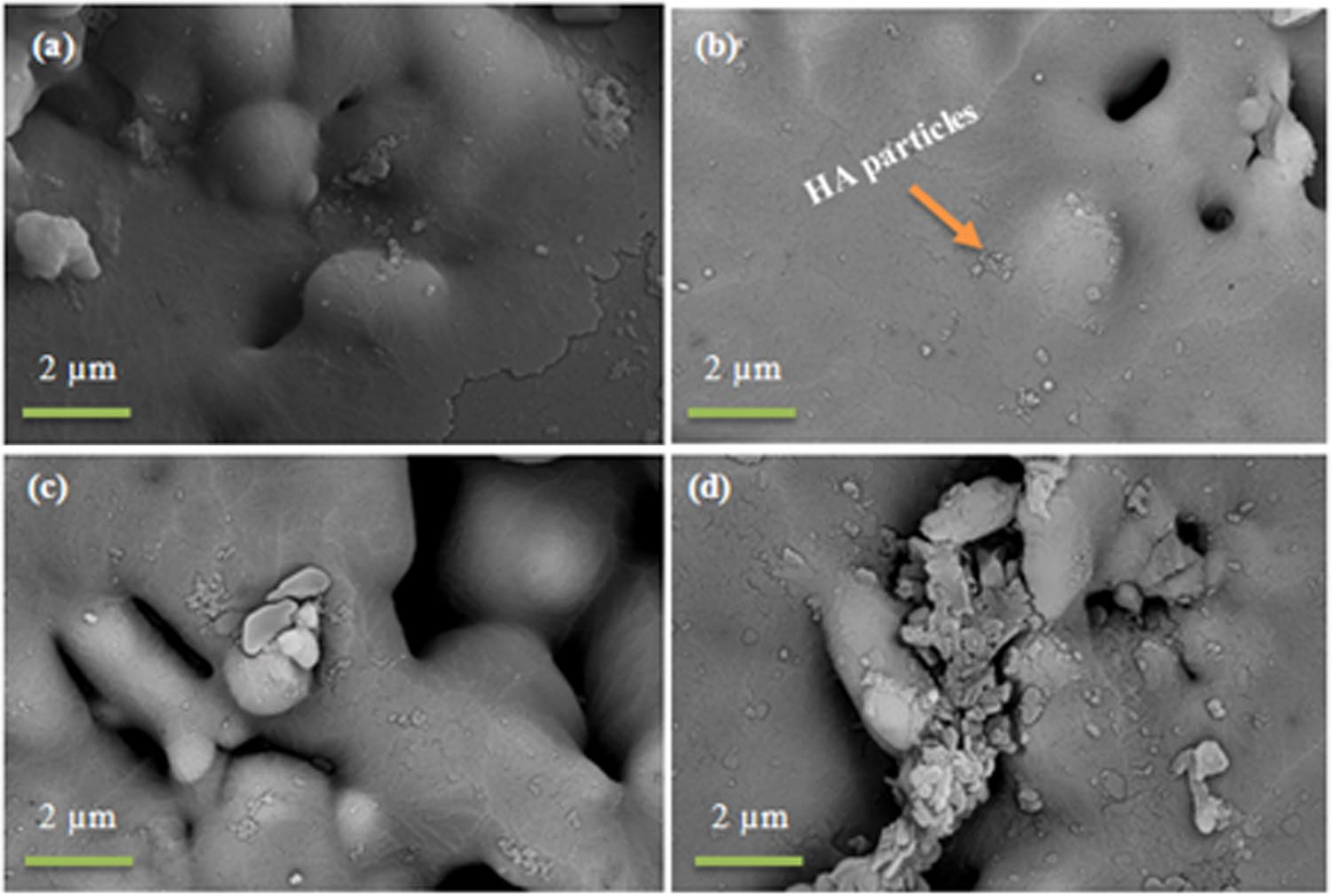
SEM images of the surface of P40B10–60 vol. % NH_4_ (HCO_3_) bioactive glass scaffolds after immersion in simulated body fluid for (**a**) 24 hr (**b**) 48 hr (**c**) 48 hr and (**d**) 168 hr.

**Figure 11:**
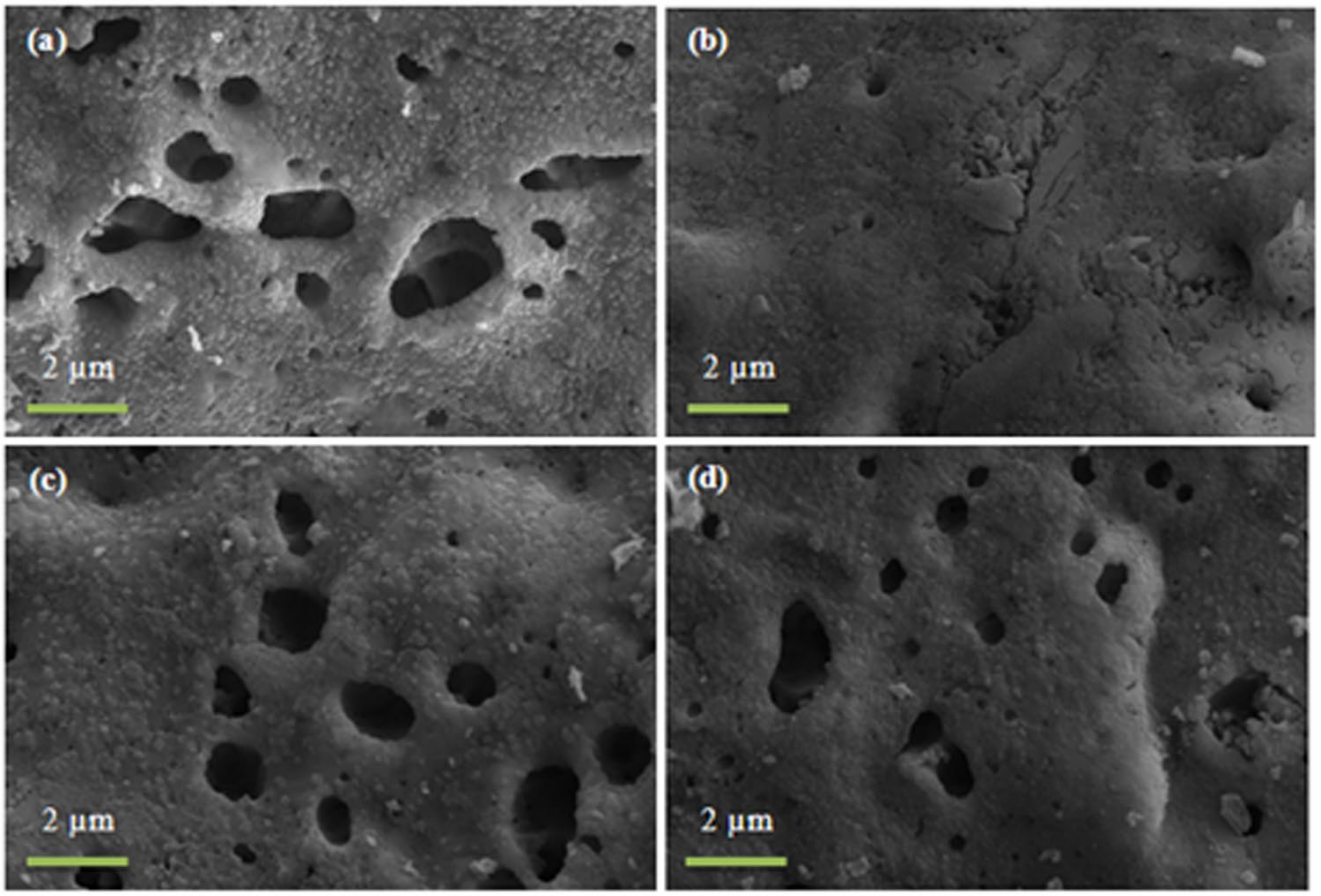
SEM images of the surface of PSr40–60 vol. % NH_4_ (HCO_3_) bioactive glass scaffolds after immersion in simulated body fluid for (**a**) 24 hr (**b**) 48 hr (**c**) 48 hr and (**d**) 168 hr.

SEM images of the glass scaffold surfaces of P40B10 and PSr40 are shown in Figs 10 and 11, respectively.

**Figure 12:**
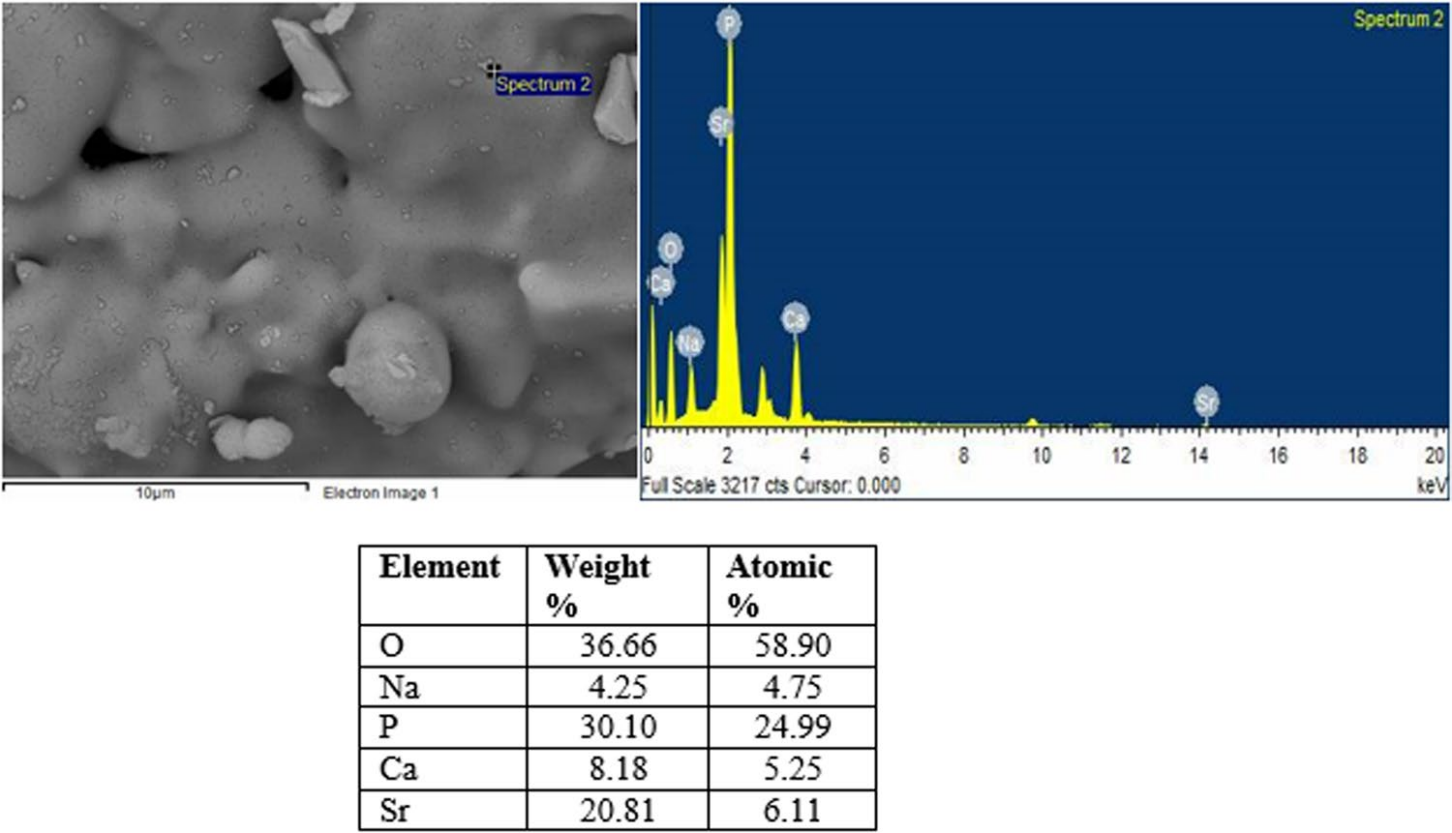
EDS spot analysis of HA particles on glass P40B10–70 vol. % NH_4_ (HCO_3_) after immersion in simulated body fluid for 168 hours.

**Figure13:**
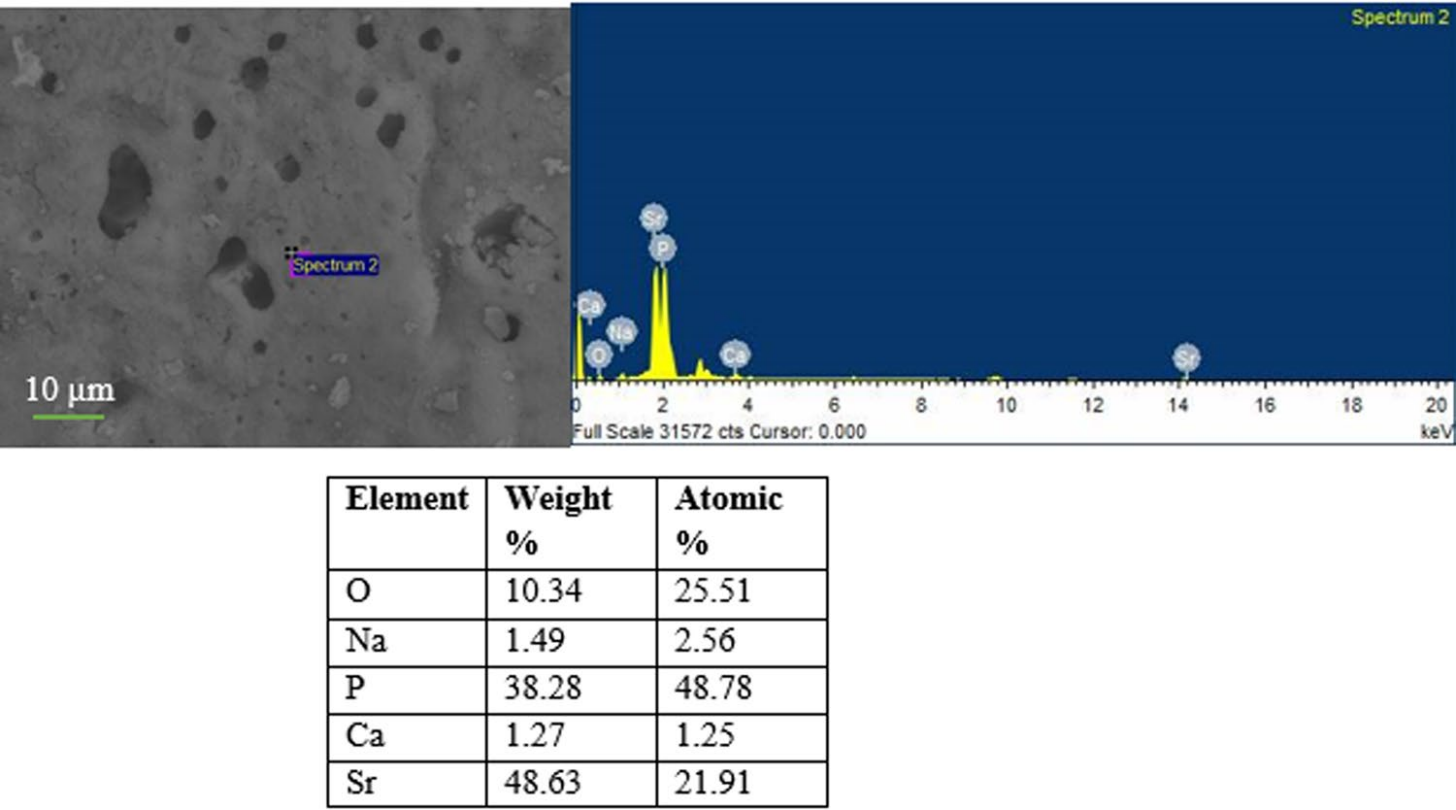
EDS spot analysis of HA particles on glass PSr40–60 vol. % NH_4_ (HCO_3_) after immersion in simulated body fluid for 168 hours.

In the case of the borophosphate scaffolds (Fig. 10); signs of a reactive layer formation could be seen. However, the surface coverage and the amount of globular particles was low. Figure 12 presents the EDS analysis of the P40B10 surface after 168hrs immersion. From the calculation of the Ca + Sr/P ratio; the ratio was found to be 0.45 which correponds to the ratio of the original glass composition (~0.5). SEM images of the phosphate scaffolds (Fig. 11), as a function of immersion time, exhibit the evolution of a surface texture which can also be attributed to a reactive layer. Figure 13 presents the EDS analysis of the HA particles formed on the glass surface of Sr after immersion for 168 hours. EDS analysis reveal the presence of small proportion of Ca at the surface of the scaffolds. The (Ca + Sr)/P also increased from 0.40 in the original glass to 0.47 at the surface of the scaffolds immersed for 168 hours.

#### Compressive strength

**Figure 14:**
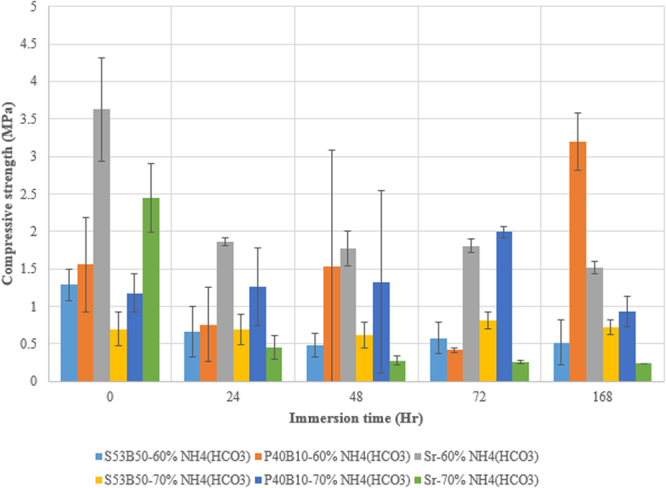
Compressive strength of scaffolds as a function of immersion time in simulated body fluid at 60 and 70 vol. % content.

The compressive strength of the scaffolds before and after immersion in SBF are presented in Fig. 14 for scaffolds processed with 60 and 70% porogens. Before immersion it appears clear that the scaffolds processed with 70% porogens exhibit lower compressive strength than the ones processed with 60%. All the scaffolds processed with 60% porogens, exhibit a decrease in their compressive strength at 24 hours immersion. At longer immersion time, the compressive strength remain constant, except for the P40B10 scaffolds, which exhibited a rise in mechanical properties. S53B50 and P40B10 processed with 70% porogens seem to remain constant as a function of immersion time, whereas the PSr40 scaffolds see their compressive strength decreasing.

## Discussion

Scaffolds from the S53B50 glass compositions were obtained without any crystallization as shown in Fig. 1. The increase in pH upon immersion of the borosilicate scaffolds (Fig. 2a), in SBF, was expected from previous research^25–27^. The increase in pH is generally attributed to ion exchange and leaching of alkaline and alkaline earth ions, typical of the non-congruent dissolution of silicate and borosilicate glasses. Furthermore, one should keep in mind that the presented results are obtained without refreshing the immersion solution. This leads to high pH (pH 9). In the body, the fluid circulates and therefore can mitigate the increase in pH. However, it is important to keep in mind that excessive rise in pH can have a negative impact on osteogenesis^28^. The dissolution of the glass was further confirmed by the significant weight loss that the scaffolds exhibited with respect to immersion time (Fig. 3a) and from release of [B], [Ca] and [Si] (Fig. 4). In Fig. 4c, one can see that the [P] content decreases with time. This is typically correlated to the precipitation of a reactive layer. EDS/SEM and XRD were employed to evidence changes in the surface composition and/or morphology. XRD revealed a crystalline phase present in small amount already at 24 h of immersion (Fig. 7a), the increase in the diffraction peak intensity indicates that the crystals density increases. However, as only few diffraction peak are seen it is difficult to ascertain the crystalline phase. Nonetheless, diffraction peaks, in this region, upon immersion of bioactive glass in SBF are usually correlated with apatite formation. SEM imaging revealed the precipitation of particles with a globular shape (Fig. 8). The number of particles are indeed increasing with increasing immersion time. EDX analysis on these small globular crystals were found to have a Ca/P ratio of 1.65 which is close to the 1.67 typical for hydroxyapatite. These crystals were not only found at the surface of the scaffolds but also within the pores at the centre of the scaffolds indicating that the fluid circulation was sufficient to induce HA precipitation within the 3D construct.

Borophosphate and phosphate scaffolds were also prepared. While amorphous scaffold could be obtained from the borophosphate glass composition, all scaffolds obtained from the phosphate glass showed heavy crystallization (Fig. 1). This can be attributed to the surface crystallization reported in^12^ for the PSr40 glass. Due to the small size of the particles the surface crystallization is further enhanced. The borophospahte glass was developed to increase the stability of the glass to crystallization. While, ultimately, amorphous scaffolds were targeted, studying fully crystallized phosphate scaffolds can help understand the impact of crystallization on the glass’ bioactivity. The scaffolds were immersed in SBF for up to 168 hours. Both composition are known to exhibit congruent dissolution as opposed to the non-congruent dissolution of silicate glasses^29^. This is known to lead to substantial amount of phosphate within the media. The phosphate release upon immersion can be seen in Figs 5c and 6b and is a cause for the decrease in the pH seen in Fig. 3b and c. As expected, the borosilicate glass exhibit slower degradation than the phosphate glass^15^. This is demonstrated by (i) the higher pH, (ii) the lower mass loss and (iii) the lower [P] concentration in solution when testing the borosilicate scaffolds as opposed to the phosphate one. In both case a [Ca] consumption can be seen (Figs 5b and 6a). A [Ca] consumption is often related to the precipitation of a reactive layer. However, one should keep in mind that, in the case of phosphate and borophosphate glasses a dicalcium phosphate dehydrate layer is expected to precipitate (CaHPO_4_) as opposed to the hydroxyapatite that typically precipitate at the surface of the bioactive silicate and or borosilicate glasses^30^. From Figs 10 and 11, a change in texture can be seen with deposition of small nodules at the surface of the scaffolds. However, in the case of the borosilicate, the EDX analysis does not reveal any change in the surface composition (Fig. 9) as evidenced by the constant (Ca + Sr)/P ratio. The XRD pattern does not show any signs of crystals. This can be attributed to, either, the slow dissolution/reaction rate of this glass and/or precipitation of small and amorphous reactive layer. In the case of the Sr glass scaffolds the EDX analysis indicates a small increase in the (Ca + Sr)/P ratio from 0.4 to 0.47. The presence of Ca at the surface of the glass is a clear indication of the reactive layer precipitation. However, the layer is thin and sparsely dispersed.

The dissolution of the PSr40 glass particles and its crystallization behavior have been reported in^22^ and^12^, respectively. As particulates, a thick Sr substituted CaP reactive layer formed at 48 h. Here, at 168 h of immersion the reactive layer only started precipitating. Indeed, in^30^ the reactive layer formed at the surface a metaphosphate glass containing Ca instead of Sr was close to the composition of dicalcium phosphate dehydrate (DCPD) with (Ca)/P ratio close to 1. In^12^ the reactive layer formed was similar for the Ca and Sr containing metaphosphate glass, at the exception that Sr partly substituted Ca in the reactive layer. Here the (Sr + Ca)/P ratio is much lower than 1 (~0.47), but presence of Ca indicates that the Ca-containing reactive layer started to form. This could be attributed, partially, to higher surface area to volume ratio when testing particulates compared to scaffolds. However, the surface in contact with the liquid alone does not explain fully the change in dissolution behavior. Indeed, the amount of P being released is drastically higher in the case of the scaffolds than the particulates. In^12^ it was found that two phases crystallized, in this glass composition, upon heat treatment: Sr(PO_3_)_2_ and NaSr(PO_3_)_3_, which, like Ca(PO_3_)_2_ and NaCa(PO_3_)_3_, are sparingly soluble in water^31^. Therefore, it is possible that the main by products are produced from highly soluble remaining amorphous phase. Therefore, this confirms that crystallinity reduces or even suppresses the reactive layer precipitation and lead to high phosphate release, which would have negative effects if used in contact with cells^32^.

All scaffold produced with 70 vol. % of porogens were found to exhibit faster dissolution rate. This led to higher mass loss and ion release in solution, along with more pronounced pH changed. This was expected since an increase in the porogen content give rise to higher overall porosity, therefore increasing the surface area in contact with the solution.

### Compressive strength

The compressive strength of the as-prepared S53B50 scaffolds was 1.29 ± 0.21 MPa. In comparison, the compressive strength of the as prepared P40B10 scaffold was 1.56 ± 0.63 MPa while Sr had strength of 3.63 ± 0.69 MPa. The high strength of glass Sr might be due to the crystalline phases formed in the amorphous matrix, as it has been reported by Kauer *et al*.^33^ that crystallization enhances the mechanical properties of bioactive glass. The compressive strength falls within the lower limits of cancellous bone. The obtained mechanical strength are in line with the obtained when scaffolds. With various composition but similar porosity were produced via thermal bonding or foam replication^34^.Upon immersion for 24 hours, the strength of all scaffolds produced with 60 vol. % of porogens decreased. This decrease can be attributed to the initial dissolution of the scaffolds leading to weakening of the pore struts. At longer immersion time the mechanical strength of the S53B50 and PSr40 scaffolds stabilizes as already evidenced by Liu *et al*.^35^ with 13–93 scaffolds prepared by robotic deposition and tested *in-vitro* and *in-vivo*. The decrease in the mechanical properties, in their studies, was up to 2 weeks and then it remains constant for up to 12 weeks. The longer time before stabilization of the mechanical properties can be accounted for the lower degradation rate of this glass compared to the phosphate or borosilicate glasses studied here. In another hand the mechanical properties of the P40B10 scaffolds shows almost no change in the mechanical properties for short immersion times and even some increase in the compression strength at longer immersion times. This could be attributed to the slow degradation of this glass and to the smaller pores being blocked by debris during immersion. The mechanical properties of the scaffolds decreases, regardless of the composition, with increasing the porogens content. This is attributed to the higher overall porosity and therefore the higher density of critical flaws. However the mechanical properties exhibited similar trends, upon immersion, regardless of their porosity.

## Materials and Methods

### Glass processing

The three experimental glasses had a nominal oxide composition (Table 1) of 26.93SiO_2_-26.93B_2_O_3_-22.66Na_2_O-1.72P_2_O_5_-21.77CaO for S53B50 and (50-x) P_2_O_5_-xB_2_O_3_-20CaO-20SrO-10Na_2_O for P40B10 and Sr, where; x = 0 (Sr) and x = 10 (P40B10). The glass compositions were melted by mixing relevant proportion of analytical grade of Na_2_CO_3_, H_3_BO_3_, CaCO_3_, CaHPO_4_.2H_2_O and sand (99.4% pure SiO2) for glass S53B50; and NaPO_3_, H_3_BO_3_, SrCO_3_, CaCO_3_, (NH_4_)_2_HPO, Ca(PO_3_)_2_, Sr(PO_3_)_2_ for glass P40B10 and Sr. The mixtures were then melted in air in a platinum crucible at temperature from 1100 °C to 1250 °C for glasses P40B10, Sr and S53B50, respectively. The melt was poured onto a graphite mould and annealed overnight at 40 below their respective Tg and then allowed to slowly cool to room temperature in the annealing furnace. The obtained rods were then crushed to powders with particle size <75 µm.

**Table 1:** Nominal (and measured) composition of S53B50, P40B10 and PSr40 glasses presented in molar percent (mol. %).

Composition (mol. %)	S53B50	P40B10	PSr40
SiO_2_	26.93 (26.1)	0	0
Na_2_O	22.66 (23.2)	10.00 (11.5)	10.00 (11.1)
CaO	21.77 (22.55)	20.00 (19.0)	0
B_2_O_3_	26.93 (26.7)	10.00 (9.8)	0
P_2_O_5_	1.72 (1.5)	40.00 (38.8)	50.00 (48.7)
SrO	0	20.00 (20.9)	40.00 (40.2)

### Scaffold preparation

The scaffolds were produced by adding NH_4_ (HCO_3_) foaming agent which was burned out at 67 °C to create pores into the material. The S53B50 and P40B10 scaffolds were sintered at 540 °C and the PSr40 scaffolds at 500 °C. The sintering temperatures at which the scaffolds were created was based on the sintering behavior of the glasses that showed no or minor crystallization as observed from the XRD analysis reported previously. Sintering was conducted in air in a muffle furnace at a heating rate of 10 °C/min for 1 hour at the sintering temperature.

### *In vitro* evaluation

To assess the *in vitro* bioactivity of the scaffolds, the scaffolds were immersed in simulated body fluid (SBF) solution at 37 °C as proposed by^36^. The SBF solution mimics the ionic composition of the blood plasma and the ion concentration of the solutions are summarized in Table 2. The simulated body fluid was prepared using 7.996 g NaCl, 350 mg NaHCO_3_, 224 mg KCl, 228 mg K2HPO_4_*3H_2_O, 305 MgCl_2_*6H_2_O, 40 ml 1 N HCl acid, 368 mg CaCl_2_*2H_2_O, 71 mg Na_2_SO4 and 6.057 g tris (hydroxymethyl) amino methane, (CH_2_OH)_3_CNH_2_, (Tris) per litre SBF using deionized water. The pH was adjusted to 7.40 using 1 N hydrochloric acid with the aid of a pH meter.

**Table 2:** Comparison of the ion concentrations of SBF K9 with those of blood plasma.

Ion Conc. (mM)	Na^+^	K^+^	Mg^2+^	Ca^2+^	Cl^-^	(HCO_3_)^−^	(HPO_4_)^2−^	SO_4_^2−^
SBF	142.0	1.5	1.5	2.5	147.8	4.2	1.0	0.5
Blood plasma	142.0	1.5	1.5	2.5	103.0	27.0	1.0	0.5

The mass of sample to volume of SBF ratio (40 mg/1 ml) was kept constant for all the experiments. About 19–25 ml SBF was measured into 50 ml PP conical tubes and 900–1070 mg of scaffolds were dispersed in the solution for 24, 48, 72 and 168 hours at 37 °C in an incubating shaker (HT Infors Multitron) at an agitation speed of 100 rpm. The experiments were conducted in duplicate. At the end of each time period, the change in the solution pH was measured and 5 ml was measured from the solution which was then diluted with deionised water. 15 ml was measured into a 15 ml PP conical tube and stored at 4 °C for inductively coupled Plasma-Optical emission spectroscopy analysis. After testing, the scaffolds were removed from the solution, rinsed with ethanol to stop any further reaction, dried at 37 °C for SEM-EDS and XRD analysis

### Characterization

Inductively Coupled Plasma – Optical Emission Spectroscopy was performed on immersion liquid diluted 10X except for the Sr scaffolds which were diluted 100X, due to the large amount of P. Na was not quantified due to the high Na content in SBF leading to high inaccuracy. The emission lines used were 253.56 nm for P; 393,366 nm for Ca; 421.552 nm for Sr; 249.772 nm for B; and 251.611 nm for Si. The top surface of the scaffolds were analysed using SEM-EDS and XRD to evidence the formation of hydroxyapatite after immersion in SBF.

### Mechanical properties

The compressive strength of the scaffolds (17 mm in diameter × 4 mm) as prepared and after immersion in the SBF for the selected durations, was measured using an Instron testing machine (Model 1175-K5942; Instron, Advanced laboratory solutions, SA) at a crosshead speed of 0.5 mm/min. For the scaffolds immersed in SBF, the compressive strength was measured after drying the scaffolds. Two samples were tested for the samples that were immersed in SBF for each duration, and the mean strength and standard deviation were determined. Ten samples were tested to determine the compressive strength of the scaffolds before they were immersed in SBF.

## Conclusion

This study demonstrates that control over the crystallization of the glass upon sintering is of crucial importance and that all scaffolds should be carefully evaluated, in terms of bioactivity and ion release, post-processing. Crystallization of a bioactive phosphate glass during the scaffold processing leads to uncontrolled phosphate release that can be harmful to living cells while improving the construct mechanical properties.

Borosilicate and borophospahte bioactive glass scaffolds can be processed while maintaining the amorphous nature of the construct. Such scaffolds show predictable reaction *in vitro* based on known data from particulates. As expected the borophosphate scaffolds show slow dissolution within the testing period while the borosilicate glass scaffolds precipitated a reactive layer (HA) at their surfaces.

The large overall porosity (>60%), large pore size (>200 µm), good pore interconnection, and mechanical properties closed to cancellous bone combined with the control ion release and predictable dissolution rate of the borosilicate and borophosphate glass scaffolds makes them good candidate for further testing in contact with living cells.
